# Evolution of the Yields and Composition of Essential Oil from Portuguese Myrtle (*Myrtus comunis* L.) through the Vegetative Cycle

**DOI:** 10.3390/molecules14083094

**Published:** 2009-08-20

**Authors:** Paula C. Pereira, Maria-João Cebola, M. Gabriela Bernardo-Gil

**Affiliations:** 1 Centre for Biological and Chemical Engineering, IBB; DEQB, IST, Av. Rovisco Pais, 1049-001 Lisboa, Portugal; E-mails: paulapereira1@sapo.pt (P.P), m.joao.cebola@gmail.com (M.J.C.); 2 Faculdade de Engenharias e Ciências Naturais, ULHT, Av. Campo Grande, 376, 1749-024 Lisboa, Portugal; E-mails: paulapereira1@sapo.pt, m.joao.cebola@gmail.com

**Keywords:** myrtle, *Myrtus communis* L., essential oil, limonene, 1,8-cineole, myrtenyl acetate

## Abstract

The chemical composition of the essential oil of Portuguese myrtle was determined at different developmental stages of the plant: pre-flowering, flowering, unripe and ripe berries. The oil was extracted separately by Clevenger distillation from leaves, branches and berries. The yields vary from 0.33% to 0.74% for leaves, 0.02% to 0.19% for branches, and 0.11% to 0.23% for berries. The highest yields were obtained for the leaves in October, and for the berries in September; branches show similar values in the months of June, July and September, and the samples collected in May and October produced very little amount of oil. Altogether, September seems to be the month with the best yields for the three parts of the plant. The essential oils were analyzed by GC and GC/MS, and a total of thirty five components were identified. The major components were limonene+1,8-cineole [25.9% (berries)–39.5% (leaves)], myrtenyl acetate [6.6% (berries)–24.8% (leaves)], α-pinene [9.7% (berries)–21.5% (leaves)], and linalool [6.2% (leaves)–36.5% (berries)]. Portuguese myrtle belongs to the group of myrtles which are characterized by the presence of myrtenyl acetate as one of the major components.

## 1. Introduction

Myrtle (*Myrtus communis* L.) is an evergreen shrub, belonging to the family of Myrtaceae, typical of the Mediterranean flora. In Portugal, myrtle grows wild mainly in the central and southern parts of the country. Its leaves are very fragrant, which is the reason for the extensive use of the plant in the perfumery and cosmetic industries. It is traditionally used as antiseptic, disinfectant, and hypoglycemic agent [1]. Different parts of the plant find various uses in the food industry, such as flavoring for meat and sauces [2]. In Corsica (France), several different products are made from myrtle like liqueur, wine, vinegar, jam, etc. [3]. The chemical composition of myrtle essential oil has been extensively studied in the Mediterranean region, such as in Spain [4], Corsica (France) [3], Albania [5], Turkey [6], Greece [7,8], Croatia [9], Morocco [10], Tunisia [11,12,13], and Sardinia (Italy) [14,15]. This, however, has not happened in relation to Portuguese myrtle, so there is a scarcity of data relating to its composition.

Myrtle oils can be separated into two groups, depending on the content in myrtenyl acetate [3]. Chalchat *et al.* [2], who analyzed myrtle leaf essential oil from seven Mediterranean regions, refers the strong variability on chemical composition of the oil, reporting the presence of myrtenyl acetate in some samples and the lack of it in some others.

The aim of our research was to address the lack of data concerning Portuguese myrtle essential oil composition, and to acquire valuable information about the best harvesting period according to the concentration of the claimed component. In the present work we studied the composition of the essential oil from three different plant materials: leaves, berries and branches over a period of time that covers the principal stages of the plant’s vegetative cycle, namely pre-flowering, flowering, unripe fruit and ripe fruit. Thus, the months covered by this study were May (pre-flower), June (flower buds), July (flower blossom), September (unripe fruit) and October (ripe fruit). The essential oil was obtained by hydrodistillation using a Clevenger type apparatus.

## 2. Results and Discussion

The essential oil yields obtained in this work are presented in Table 1. The values ranged from 0.33% to 0.74% for leaves, from 0.02% to 0.19% for branches, and from 0.11% to 0.23% for berries. The highest yields were obtained for the leaves in October and for the berries in September. The branches show similar values in the months of June, July and September. The samples collected in May and October produced very little oil. 

Altogether, September seems to be the month with the best yields for the three parts of the plant. These results disagree with the ones obtained by Jamoussi *et al*. [12], who report maximum yields at the flowering stage for Tunisian myrtle oils, while for us it was near the ripening of the fruit stage. On the other hand, Bradesi *et al*. [3] recommend the period from June to November as the best harvest time for commercial production of the essential oil.

**Table 1:** Essential oil yields for the harvested months.

Month	Stage	Yield(g/100 g dried leaves)		
		Leaves	Branches	Berries
May	Pre-flower	0.33±0.03	0.02±0.01	-
June	Flower buds	0.60±0.04	0.19±0.01	-
July	Flower blossom	0.48±0.01	0.17±0.03	-
September	Unripe fruit	0.56±0.05	0.18±0.01	0.23±0.04
October	Ripe fruit	0.74±0.09	0.07±0.01	0.11±0.06

The results obtained for the composition of the essential oil extracted from leaves, branches and berries are shown in Table 2, Table 3 and Table 4, respectively. Thirty five components were identified, representing 93.5–95.4% of the total composition for leaves, 80.3–88.1% for branches, and 91.3–93.8% for berries. All three parts of the plant show a high content in monoterpenes and esters. The leaves exhibit a particularly high content in monoterpenes with a peak at the flowering stage (July) and a minimum in May, while the esters, the second major group, reaches a peak in May and is at a minimum in July. As for the berries, the highest content in monoterpenes happens when they are ripe, while the unripe berries show a higher content in esters. The essential oil yields obtained for the branches were very low, especially in May.

**Table 2:** Composition of the leaves essential oil of Portuguese myrtle.

Components ^a^	LRI ^b^	ID Method ^c^	Composition (w/w%) ± SD ^e^				
			May	June	July	September	October
Tricyclene^d^	908	MS, LRI	0.08±0.00	0.12±0.00	0.08±0.00	0.14±0.00	0.19±0.00
*α* -Thujene^d^	917	MS, LRI	0.05±0.00	0.08±0.00	0.10±0.00	0.06±0.00	0.05±0.00
*α*-Pinene	922	MS, LRI, std	10.38±0.07	16.45±0.01	21.50±0.10	13.12±0.22	15.25±0.01
Camphene^d^	934	MS, LRI	0.03±0.00	0.03±0.00	0.04±0.00	0.03±0.00	0.03±0.01
β-Pinene	963	MS, LRI, std	0.12±0.00	0.16±0.00	0.26±0.00	0.16±0.01	0.19±0.00
Myrcene	985	MS, LRI, std	0.11±0.00	0.09±0.00	0.14±0.00	0.08±0.01	0.08±0.00
δ-3-Carene^d^	998	MS, LRI	0.02±0.00	0.12±0.00	0.05±0.00	0.18±0.00	0.02±0.00
*α*-Terpinene	1001	MS, LRI, std	0.17±0.01	0.12±0.00	0.11±0.01	0.13±0.00	0.19±0.01
p-Cymene	1014	MS, LRI, std	0.04±0.00	0.03±0.00	0.03±0.00	0.04±0.00	0.04±0.00
Limonene+1,8-Cineole	1024	MS, LRI, std	20.03±0.11	31.33±0.13	39.45±0.10	32.13±0.22	36.78±0.09
o-Cymene^d^	1037	MS, LRI	0.01±0.00	0.01±0.00	0.02±0.00	0.06±0.00	0.06±0.00
γ-Terpinene	1045	MS, LRI, std	0.21±0.00	0.11±0.00	0.11±0.00	0.18±0.00	0.15±0.00
Linalool oxide	1059	MS, LRI, std	0.04±0.00	0.20±0.00	0.30±0.00	0.05±0.00	0.05±0.00
*α*-Terpinolene^d^	1080	MS, LRI	0.10±0.00	0.20±0.00	0.25±0.01	0.09±0.00	0.07±0.00
Linalool	1096	MS, LRI, std	7.58±0.06	7.01±0.02	6.19±0.03	9.27±0.03	7.91±0.01
Fenchol	1100	MS, LRI, std	1.10±0.01	0.57±0.01	0.52±0.02	0.75±0.01	0.73±0.03
Trans-inocarveol^d^	1129	MS, LRI	0.15±0.00	0.04±0.00	0.04±0.00	0.10±0.00	0.15±0.00
Borneol	1160	MS, LRI, std	0.06±0.00	0.08±0.00	0.14±0.00	0.08±0.00	0.08±0.01
Terpinen-4-ol	1169	MS, LRI, std	0.19±0.00	0.22±0.00	0.29±0.00	0.25±0.00	0.25±0.00
*α*-Terpineol	1183	MS, LRI, std	2.69±0.03	3.63±0.01	5.15±0.02	3.34±0.01	3.46±0.00
Myrtenol	1188	MS, LRI, std	2.86±0.07	0.79±0.00	0.32±0.00	1.94±0.01	1.85±0.00
Geraniol	1252	MS, LRI, std	0.89±0.00	0.84±0.01	0.89±0.00	0.80±0.01	0.94±0.00
Borneol acetate	1278	MS, LRI, std	0.02±0.01	0.012±0.00	0.02±0.00	0.01±0.00	0.02±0.00
Trans pinocarvyl acetate^d^	1292	MS, LRI	0.64±0.00	0.39±0.01	0.16±0.01	0.46±0.05	0.39±0.00
Myrtenyl acetate	1323	MS, LRI, std	37.62±0.13	22.19±0.09	7.40±0.02	24.83±0.21	20.75±0.02
*α*-Terpenyl acetate^d^	1336	MS, LRI	0.33±0.00	0.30±0.00	0.22±0.00	0.19±0.00	0.23±0.00
Eugenol^d^	1343	MS, LRI	0.33±0.01	0.84±0.00	1.54±0.01	0.49±0.00	0.09±0.00
Neryl acetate	1363	MS, LRI, std	0.06±0.00	0.09±0.00	0.12±0.02	0.05±0.00	0.04±0.00
Geranyl acetate	1376	MS, LRI, std	0.38±0.00	0.24±0.00	0.11±0.00	0.21±0.00	0.14±0.00
Methyl eugenol^d^	1381	MS, LRI	2.13±0.01	3.52±0.01	4.26±0.02	2.18±0.03	1.71±0.00
β-Caryophyllene	1402	MS, LRI, std	2.57±0.01	2.87±0.01	2.18±0.01	1.73±0.01	1.83±0.00
*α*-Humulene	1442	MS, LRI, std	0.88±0.00	0.51±0.01	0.84±0.01	0.26±0.01	0.17±0.00
Geranyl isobutyrate^d^	1513	MS, LRI	0.92±0.05	1.10±0.03	0.98±0.01	0.83±0.03	0.75±0.01
Caryophyllene oxide	1571	MS, LRI, std	0.33±0.01	0.18±0.00	0.27±0.02	0.36±0.01	0.39±0.00
Humulene oxide^d^	1597	MS, LRI	0.38±0.05	0.29±0.00	0.31±0.01	0,41±0.01	0.40±0.01
Monoterpenes			31.3	48.8	62.1	46.3	53.1
Alcohols			15.8	14.0	15.1	17.0	15.5
Esters			39.9	24.3	9.0	26.6	22.3
Ethers			2.1		4.3	2.2	1.7
Sesquiterpenes			3.8	3.6	3.3	2.4	2.4
Oxides			0.7	0.7	0.9	0.8	0.8
Identified compounds			93.4	94.7	94.4	94.9	95.4

^a^ Compounds are listed in order of their elution on a HP-5 column; ^b^ Linear retention indices as determined on a DB-5MS column using a homologous series of n-alkanes; ^c^ Methods of identification: MS, by comparison of the mass spectrum with those of the computer mass libraries; LRI, by comparison of LRI with those from the literature; std, by injection of the authentic sample; ^d^ Tentatively identified according of the mass spectrum (MS) and by comparison of LRI with the linear retention time; ^e^ Standard deviation.

**Table 3:** Composition of the branches essential oil of Portuguese myrtle.

Components^a^	LRI^b^	ID Method^c^	Composition (%)±SD^e^				
			May	June	July	September	October
Tricyclene^d^	908	MS, LRI		0.01±0.00	0.01±0.00	0.02±0.00	
*α*-Thujene^d^	917	MS, LRI		0.05±0.00	0.03±0.00	0.12±0.00	
*α*-Pinene	921	MS, LRI, std		6.55±0.01	4.22±0.22	8.81±0.61	2.78±0.77
Camphene^d^	937	MS, LRI		0.01±0.00	0.01±0.00	0.05±0.00	
β-Pinene	963	MS, LRI, std		0.11±0.00	0.08±0.00	0.34±0.01	
Myrcene	986	MS, LRI, std		0.08±0.00	0.08±0.02	0.46±0.02	
δ-3-Carene^d^	998	MS, LRI		0.05±0.00	0.04±0.01	0.16±0.00	
*α*-Terpinene	1003	MS, LRI, std		0.09±0.00	0.08±0.01	0.25±0.00	
p-Cymene	1015	MS, LRI, std		0.07±0.00	0.03±0.00	0.82±0.05	
Limonene+1,8-Cineole	1022	MS, LRI, std		11.85±0.00	12.04±0.31	20.40±2.54	10.58±0.77
o-Cymene^d^	1036	MS, LRI		0.01±0.00	0.03±0.00	0.03±0.00	
γ-Terpinene	1043	MS, LRI, std		0.25±0.00	0.09±0.00	1.19±0.07	
Linalool oxide	1051	MS, LRI, std		0.14±0.00	0.08±0.01	0.26±0.00	
*α*-Terpinolene^d^	1081	MS, LRI		0.22±0.00	0.12±0.00	0.20±0.01	
Linalool	1097	MS, LRI, std		4.59±0.01	10.47±0.18	4.24±0.14	3.42±0.52
Fenchol	1100	MS, LRI, std		0.71±0.03	0.48±0.07	0.72±0.10	
Trans-pinocarveol^d^	1129	MS, LRI		0.09±0.02	0.10±0.00	0.18±0.00	
Borneol	1160	MS, LRI, std		0.21±0.00	0.19±0.00	0.27±0.01	
Terpinen-4-ol	1169	MS, LRI, std		0.28±0.02	0.32±0.01	0.70±0.03	
*α*-Terpineol	1184	MS, LRI, std		3.38±0.01	5.76±0.08	2.70±0.20	2.33±0.41
Myrtenol	1189	MS, LRI, std	44.03±7.00	3.05±0.00	8.38±0.21	6.78±0.77	12.41±2.02
Geraniol	1255	MS, LRI, std		1.16±0.00	2.83±0.06	1.85±0.07	3.17±0.08
Borneol acetate	1282	MS, LRI, std		0.02±0.00	0.04±0.00	0.09±0.03	0.76±0.02
Trans pinocarvyl acetate^d^	1292	MS, LRI		0.71±0.03	0.56±0.00	0.55±0.03	0.76±0.02
Myrtenyl acetate	1322	MS, LRI, std	23.97±6.89	33.55±0.08	24.62±0.21	21.97±0.64	26.25±4.03
*α*-Terpenyl acetate^d^	1337	MS, LRI		1.47±0.01	1.53±0.00	0.95±0.08	0.62±0.38
Eugenol^d^	1343	MS, LRI, std		1.66±0.00	1.49±0.01	1.23±0.75	
Neryl acetate	1364	MS, LRI, std		0.21±0.01	0.05±0.00	0.24±0.01	
Geranyl acetate	1377	MS, LRI, std		0.43±0.00	0.23±0.01	0.18±0.00	
Methyl eugenol^d^	1383	MS, LRI, std		5.16±0.01	3.66±0.01	4.93±0.24	6.53±0.24
*β*-Caryophyllene	1404	MS, LRI, std		5.34±0.02	4.48±0.09	2.85±0.09	2.56±088
*α*-Humulene	1442	MS, LRI, std	19.12±5.02	1.57±0.00	0.66±0.00	1.48±0.08	0.88±0.31
Geranyl isobutyrate^d^	1513	MS, LRI		0.85±0.03	0.88±0.01	0.10±0.01	
Caryophyllene oxide	1573	MS, LRI, std		0.66±0.00	0.88±0.03	1.67±0.16	6.04±0.22
Humulene oxide^d^	1599	MS, LRI, std		0.84±0.07	0.92±0.09	1.29±0.13	2.00±0.81
Monoterpenes				19.3	16.8	32.8	13.4
Alcohols			44	15.1	30.1	18.7	20.9
Esters			24	37.2	27.91	24.1	27.4
Ethers				5.2	3.7	4.9	7.0
Sesquiterpenes			19	7.6	6.0	6.0	9.5
Oxides				1.6	1.9	3.2	8.0
Identified compounds			87	85.4	83.4	88.1	80.2

^a^ Compounds are listed in order of their elution on a HP-5 column; ^b^ Linear retention indices as determined on a DB-5MS column using a homologous series of n-alkanes; ^c^ Methods of identification: MS, by comparison of the mass spectrum with those of the computer mass libraries; LRI, by comparison of LRI with those from the literature; std, by injection of the authentic sample; ^d^ Tentatively identified according of the mass spectrum (MS) and by comparison of LRI with the linear retention time; ^e^ Standard deviation.

**Table 4:** Composition of the berries essential oil of Portuguese myrtle.

Components^a^	LRI^b^	ID Method^c^	Composition (%)±SD^e^	
			September	October
Tricyclene^d^	911	MS, LRI	0.02±0.00	0.05±0.00
α- Thujene^d^	919	MS, LRI	0.023±0.00	0.04±0.00
α-pinene	923	MS, LRI, std	4.08±0.36	9.65±0.02
Camphene^d^	943	MS, LRI	0.01±0.00	0.01±0.00
β-Pinene	965	MS, LRI, std	0.09±0.01	0.17±0.00
Myrcene	992	MS, LRI, std	0.04±0.02	0.07±0.01
δ-3-Carene^d^	1003	MS, LRI	0.14±0.01	0.02±0.01
α-Terpinene	1010	MS, LRI, std	0.01±0.01	0.13±0.01
p-cymene	1020	MS, LRI, std	0.11±0.01	0.11±0.00
Limonene+1,8-cineole	1025	MS, LRI, std	21.02±0.88	25.28±0.09
o-Cymene^d^	1040	MS, LRI	0.01±0.00	
γ-Terpinene	1053	MS, LRI, std	0.21±0.01	0.16±0.01
Linalool oxide	1071	MS, LRI, std	0.02±0.00	0.02±0.00
α-Terpinolene^d^	1083	MS, LRI,	0.14±0.01	0.07±0.01
Linalool	1098	MS, LRI, std	7.50±0.33	6.56±0.07
Fenchol	1102	MS, LRI, std	0.57±0.04	0.65±0.03
Trans pinocarveol^d^	1130	MS, LRI	0.13±0.03	0.17±0.04
Borneol	1162	MS, LRI, std	0.13±0.01	0.10±0.01
Terpinen-4-ol	1171	MS, LRI, std	0.38±0.02	0.26±0.00
α-Terpineol	1186	MS, LRI, std	4.81±0.22	4.01±0.01
Myrtenol	1191	MS, LRI, std	3.11±0.15	3.24±0.02
Geraniol	1255	MS, LRI, std	1.02±0.06	0.93±0.00
Borneol acetate	1280	MS, LRI, std	0.02±0.00	0.06±0.07
Trans pinocarvyl acetate^d^	1294	MS, LRI	0.46±0.39	0.64±0.00
Myrtenyl acetate	1327	MS, LRI, std	36.48±1.48	32.86±0.21
α-Terpenyl acetate^d^	1338	MS, LRI	0.81±0.03	0.54±0.00
Eugenol^d^	1346	MS, LRI	1.19±0.04	0.26±0.00
Neryl acetate	1369	MS, LRI, std	0.07±0.01	0.07±0.00
Geranyl acetate^d^	1379	MS, LRI, std	0.24±0.01	0.19±0.00
Methyl eugenol	1384	MS, LRI	4.22±0.15	3.50±0.03
β-Caryophyllene	1404	MS, LRI, std	1.79±0.05	1.79±0.04
α-Humulene	1445	MS, LRI, std	0.56±0.03	0.34±0.00
β-Selinene^d^	1478	MS, LRI	0.17±0.01	0.13±0.00
α-Selinene^d^	1487	MS, LRI	0.18±0.01	0.12±0.01
Geranyl isobutyrate^d^	1513	MS, LRI	0.35±0.01	0.319±0.004
Caryophyllene oxide	1574	MS, LRI, std	0.58±0.03	0.669±0.007
Humulene oxide^d^	1601	MS, LRI, std	0.61±0.03	0.61±0.03
Monoterpenes			25.9	35.8
Alcohols			18.8	16.2
Esters			38.4	34.7
Ethers			4.2	3.5
Sesquiterpenes			3.3	3.0
Oxides			1.2	1.3
Identified compounds			91.3	93.9

^a^ Compounds are listed in order of their elution on a HP-5 column; ^b^ Linear retention indices as determined on a DB-5MS column using a homologous series of n-alkanes; ^c^ Methods of identification: MS, by comparison of the mass spectrum with those of the computer mass libraries; LRI, by comparison of LRI with those from the literature; std, by injection of the authentic sample; ^d^ Tentatively identified according of the mass spectrum (MS) and by comparison of LRI with the linear retention time; ^e^ Standard deviation.

**Figure 1:**
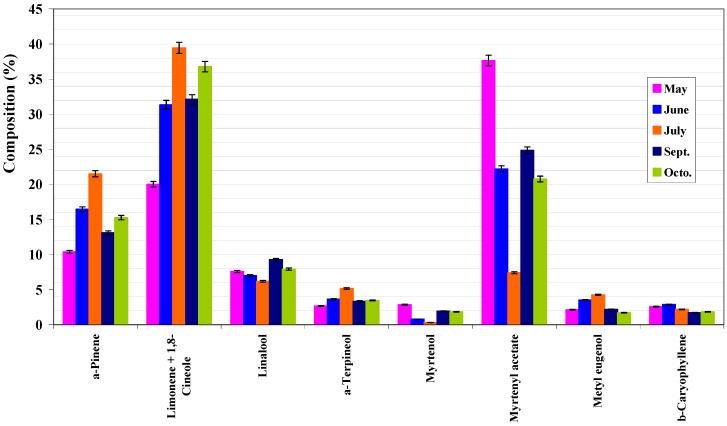
Composition of main compounds of leaves essential oil of Portuguese myrtle.

**Figure 2:**
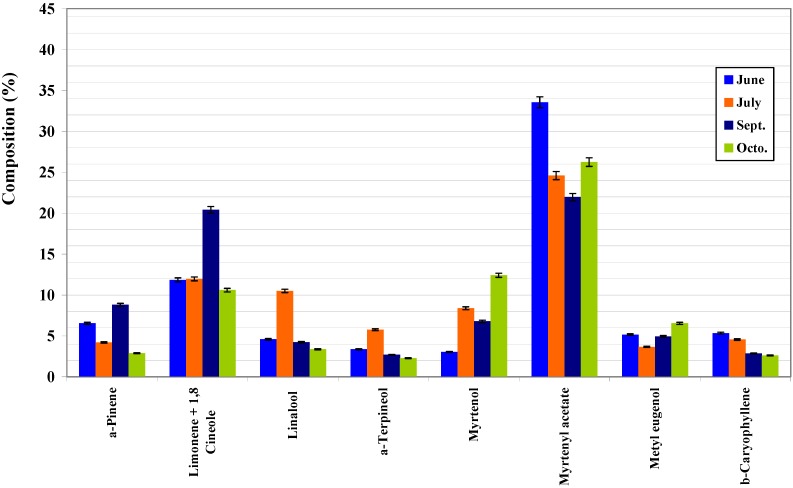
Composition of main compounds of branches essential oil of Portuguese myrtle.

**Figure 3:**
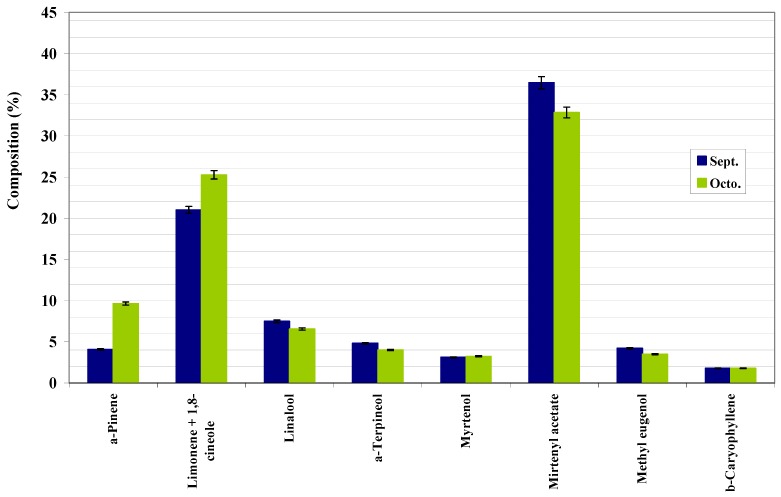
Composition of main compounds of berries essential oil of Portuguese myrtle.

From Table 2, Table 3, Table 4, and Figure 1, Figure 2, Figure 3 one may conclude that Portuguese myrtle essential oil is characterized by a high content in myrtenyl acetate and limonene + 1.8-cineole as major components. Linalool and α-pinene were also detected in appreciable quantities. All three parts of the plant show the same components, in varying proportions. These results demonstrate that Portuguese myrtle belongs to the first group [12], characterized by the presence of myrtenyl acetate as a major component. Figure 1 shows the variation of the composition of the leaves with the vegetative cycle. Two patterns stand out: a group of major components (α-pinene, limonene+1,8-cineole, α-terpineol, eugenol and methyl eugenol) shows a peak in their values in July (flowering stage), that decreases until October when it peaks again (although not as much as in July). Another group of major components, namely, myrtenyl acetate, myrtenol, linalool and fenchol, reach their peaks in May, and then their quantities started to come down until they reach a minimum in July and go up again in September. This pattern is followed, on the whole, by the branches and fruit compositions (Figure 2 and Figure 3).

The statistical analysis revealed that, for leaves and branches, the compositions are significantly different (p<0.001), when all the results were analyzed by two-factor ANOVA. However, the ANOVA single-factor applied to major components, and for each two months, showed that the results were not significant different at 95% level (p>0.05). Myrtenyl acetate is one of the major components that distinguishes between myrtles of different origin (Table 5). Its presence has been reported in the essential oils from Turkey [6], Croatia [9], Albania [5], Morocco [10], Spain [4], and Portugal (this work). The absence of myrtenyl acetate has been reported in countries or regions such as Tunisia [11,12], Greece [7], Sardinia (Italy) [14], and Corsica (France) [3]. Wannes *et al*. [15] found a small quantity of this component in the essential oil of myrtle berries collect in Tunisia. A more detailed comparison is difficult to perform since the stage of plant varies among the different studies reported, with only a few [9,12] presenting a study for different development stages of the plant. The plant material in these studies also varies: most works report results in dried plant material, but there is a few that reports the results obtained for fresh plant material [7,9,10,11]. 

It should also be noted that, while most of the published works report the extraction of the essential oil from plant materials collected for the purposes of the studies, the Albanian study [5] was carried out on a commercial myrtle oil. In spite of the fact that it is difficult to perform a straightforward comparison between the myrtles of different origins, due to the mentioned use of different experimental conditions, one aspect seems to be noticeable: the presence of myrtenyl acetate seems to be linked with a lower content in α-pinene. Myrtles which lack myrtenyl acetate seem to be richer in α-pinene. It is also interesting to notice that the work reported by Boelens *et al*. [4], shows that the fruit, unlike the leaf, no longer has myrtenyl acetate in its composition. In this work, myrtenyl acetate was present in all parts of the plant, with the highest content being found in the fruit.

**Table 5:** Comparison of essential oil composition of Myrtle from different countries/regions.

	May				June				July				September				September				October				October			
	leaves				leaves				leaves				leaves				berries				leaves				berries			
	α-pinene	1,8-cineole+limonene	Myrtenyl acetate	Linalool	α-pinene	1,8-cineole+limonene	Myrtenyl acetate	linalool	α-pinene	1,8-cineole+limonene	Myrtenyl acetate	linalool	α-pinene	1,8-cineole+limonene	Myrtenyl acetate	linalool	α-pinene	1,8-cineole+limonene	Myrtenyl acetate	linalool	α-pinene	1,8-cineole+limonene	Myrtenyl acetate	linalool	α-pinene	1,8-cineole+limonene	Myrtenyl acetate	linalool
Moroccan [10]	10^b^	43.5^b^	25^b^	0.3^b^																								
Italy (Sardinia) [14]																					50.0^d^	37.0^d^	-	0.2^d^	26.4^d^	26.8^d^	-	2.0^d^
Tunisia [12]									45.9	32.1																		
Croatia [9]	12.2	17.5	30.7	11.6	6.6	12.6	24.9	10.8	8	18.8	20.7	14.7	16.4	29.8	20.8	18.3	7.4	18.4		5.5	13.2	15.9	26.6	12.6	12.7	10.9		4.7
Greece [7]	18^b^	21.8^bc^		1.1^b^																								
Turkey [6]	6.4^b^	21.6^b^	14.5^b^	16.3^b^																								
Albania (Div) [5]					19.4	32.7	11.4	8.79																				
Albania(Elba) [5]					20.3	29	12.3	13.4																				
France (Corsica) [3]	57.2^a^	26.4^a^		1.6^a^	53^a^	23^a^		2.3^a^	51.3^a^	26.8^a^		2.5^a^	56.9^a^	22.8^a^		2.1^a^					47^a^	26.6^a^		2.4^a^				
Spain [4]					8.18	37.5	35.9	0.05																				
Tunisia[13]																	7.2	41.0	1.43	18.9					7.5	18.4	0.26	3.2
Portugal (this work)	10.4	20	37.6	7.6	16.5	31.3	22.2	7.0	21.5	39.5	7.4	6.2	13.1	32.1	24.8	9.3	4.1	2	7.5	36.5	15.3	36.8	20.8	7.9	9.7	25.9	6.6	32.9

^a^ Whole plant; ^b^ Fresh leaves; ^c^ Only limonene; ^d^ November.

## 3. Experimental

### 3.1. Plant material

Samples of myrtle were collected from the area of Sintra (central west coast of Portugal ─ latitude: 38° 58’ N; longitude: 9° 21’ W; altitude: 75 m; mean annual temperature: 15 °C; mean annual rainfall: 500 mm). The plant material was collected in May, June, July, September and October 2007, and was identified and deposited in the Herbarium of the Agronomy Superior Institute of the Technical University of Lisbon (code: LISI). The sample number one (May) was composed of leaves and branches; sample number two and three (June and July) were composed of leaves and the flower and branches were ground together; samples four and five (September and October) were composed of leaves, branches and fruits. Twelve individual plants were assessed. Adult and young leaves of all sizes were considered. Plant material was dried during two months out of the sun light, sealed into black bags, and kept at -4 °C. 

### 3.2. Chemicals

α-Pinene, β-pinene, myrcene, *α*-terpinene, p-cymene, limonene, 1,8-cineole, γ-terpinene, linalool oxide, linalool, fenchol, borneol, terpinen-4-ol, *α*-terpineol, myrtenol, geraniol, borneol acetate, myrtenyl acetate, neryl acetate*,* geranyl acetate, β-caryophyllene, *α*-humulene, caryophyllene oxide, alkane standard solutions (C_8_-C_20_) and (C_21_-C_40_) were from Aldrich (Germany, UK), Fluka (Switerland) or Extrasynthese (France).

### 3.3. Oil isolation

The essential oil was obtained from the hydrodistillation of ground dried plant material (100 g) using the well known Clevenger type apparatus [16]. Hydro-distillation was done during different times. In two hours, about 95% was obtained. So, after two hours of Clevenger distillation, the oil was recovered and was stored at -20 °C until analyzed.

### 3.4. GC-FID analysis

Samples were analyzed in three replicates. The analyses were carried out on a Hewlett-Packard 5890 Series II gas chromatograph equipped with a FID detector (supplied with air and hydrogen of high purity). The capillary column (5% diphenyl, 95% dimethylpolysiloxane) was a HP-5 cross-linked 5% (50 m x 0.32 mm i.d., 0.17 μm film thickness). The injector and detector were operated at 200°C and 250 °C respectively. Split mode was used at a ratio 1:20. The oven temperature was programmed as follows: 60 °C for 10 min, increased to 180 °C at 2 °C/min and held isothermally for 10 min. Nitrogen was used as the carrier gas (P=3 bar). Samples (0.1 μL) were injected with a 1 μL Hamilton micro-syringe. The essential oil samples were injected without dilution.

The components were identified by comparison with the linear retention times of standard components, and by comparison with LRI published in the literature [5,9,12,14,17,18]. The linear retention indices were determined using a homologous series of *n*-alkanes (C_9_ to C_25_). The results were confirmed by GC-MS.

### 3.5. GC-MS analysis

The analyses were carried out on a trace GC ultra and Trace DSQ – Thermo. The capillary column was a DB-5 (30 m, 0.5 mm i.d.; 0.5 μm film thickness). The injector and the interface were operated at 200 °C and 250 °C respectively. Helium was the carrier gas with a flow rate of 1 mL/min. Split mode was used at a ratio 1:30. MS conditions were as follows: ionization voltage, 70 eV; full rate; mass range 50-420 amu; ion source temperature 260 °C. The oven temperature program was as follows: 60 °C for 10 min, increased at 2 °C/min to 180 °C, increased at 10 °C/min to 200 °C, held isothermally for 30 min and increased at 10 °C/min to 240 °C. The compounds were identified through library search using the Wiley GC/MS Library, Mistdemo and MainLib.

### 3.6. Statistical analysis

For each sample, data are reported as mean ± standard deviation (n=3). One-way analysis of Variance (ANOVA) was performed on these values to determine whether they differed significantly at a 95% level. All statistical analyses were carried out by using the SPSS, version 14.0 for Windows.

## 4. Conclusions

Portuguese myrtle essential oils from different parts of the plant were obtained using Clevenger hydrodistillation over the vegetative cycle. Yields vary from 0.33–0.74% for the leaves, 0.02–0.19% for the branches, and 0.11–0.23% for the berries. Portuguese myrtle essential oils are characterized by high content of limonene+1,8-cineole (25.9 (berries)–39.5 (leaves)%), and myrtenyl acetate (6.6% (berries)–24.8% (leaves)) as the major components. α-pinene (9.7% (berries)–21.5% (leaves), and linalool (6.2% (leaves)–36.5% (berries) are also present at high content. All three parts of the plant show the same components, varying in proportions. These results indicate that Portuguese myrtle belongs to the group of myrtles which are characterized by the presence of myrtenyl acetate as one of the major components.
